# Surgical Management of Gallstone Ileus: A Clinical Case Report

**DOI:** 10.7759/cureus.20141

**Published:** 2021-12-03

**Authors:** Kyle Drinnon, Yana Puckett

**Affiliations:** 1 Surgery, Texas Tech University Health Sciences Center, Lubbock, USA; 2 Surgery, West Virginia University School of Medicine, Charleston, USA

**Keywords:** gallstone, small bowel obstruction, general surgery, gallstone ileus, abdominal pain

## Abstract

A 74-year-old female with a past medical history of hypertension, osteoarthritis, osteoporosis, bladder suspension, and right hip surgery presented with a one-day history of cramping abdominal pain, intermittent vomiting, and obstipation. Bowel obstruction was confirmed with imaging, and the decision was made to proceed to the operating room for an exploratory laparotomy. During the procedure, a gallstone was found in the terminal ileum and was removed.

## Introduction

Gallstone ileus is a late complication of gallstone disease that is characterized by an obstruction of the intestines caused by the lodging of gallstones in the lumen of the intestines. These obstructions can occur anywhere in the intestinal tract; however, it most commonly occurs in the terminal ileum due to its relatively narrow lumen and potentially less active peristalsis [1,2]. The gallstones commonly enter the gastrointestinal tract through a biliary-enteric fistula caused from inflammation due to late-stage gallstone disease [1]. Gallstone ileus more commonly occurs in the female and elderly (over 60-years-old) populations [3]. Other than being female and elderly, other risk factors include a history of cholelithiasis, large stones (larger than 2 cm), and episodes of acute cholecystitis [3]. We present a case of gallstone ileus obstructing the terminal ileum due to fistula formation between the gallbladder and the duodenum that was managed with exploratory laparotomy and fistula repair.

## Case presentation

**Figure 1:**
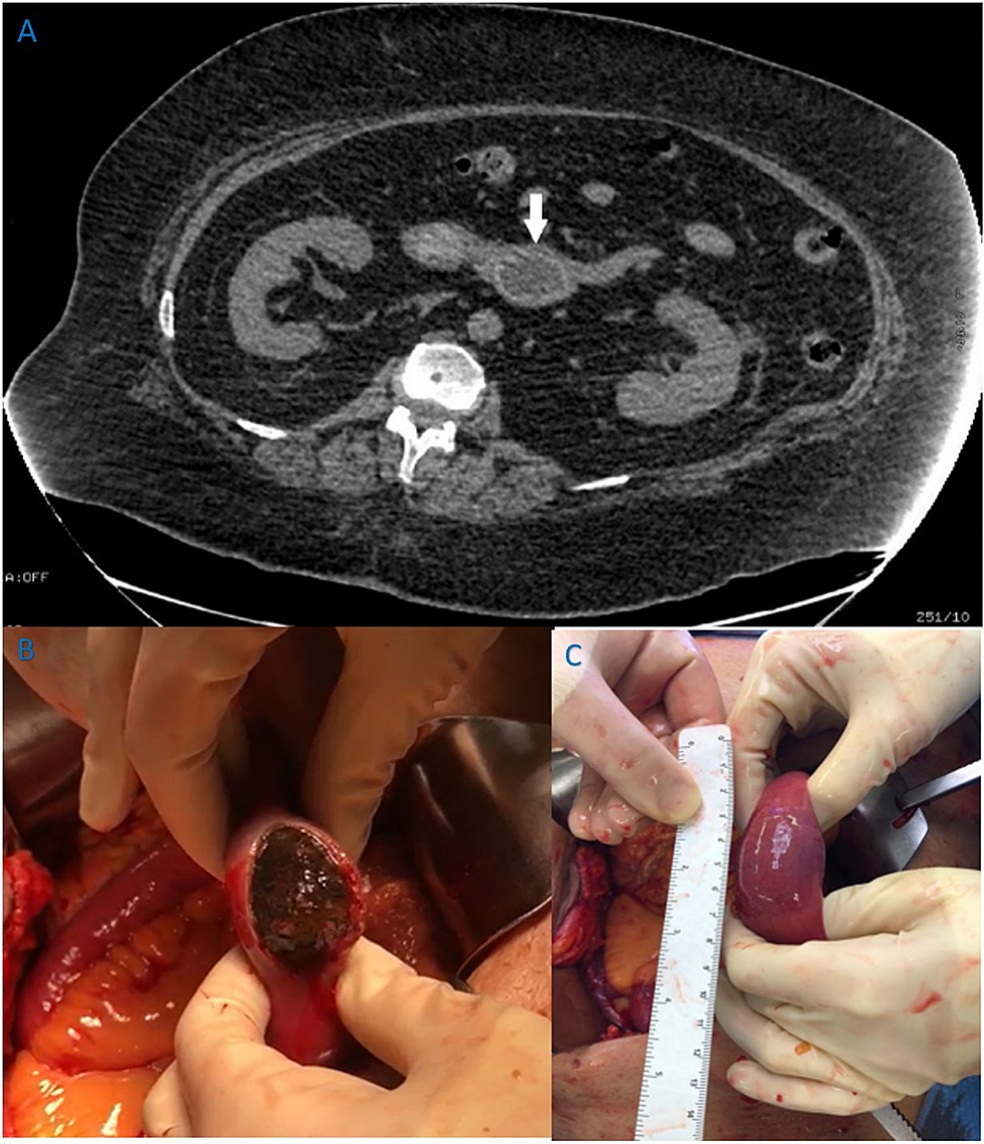
Gallstone ileus. (A) CT scan showing gallstone obstructing ileum (white arrow). (B) Obstructive gallstone in process of being gently removed from terminal ileum. (C) Impacted gallstone obstructing terminal ileum measuring roughly 5 cm × 3 cm × 4 cm found intraoperatively. CT: computed tomography.

A 74-year-old female with a past medical history of hypertension, osteoarthritis, osteoporosis, bladder suspension, and right hip surgery presented with a one-day history of cramping abdominal pain, intermittent vomiting, and obstipation. On examination, her abdomen was distended and tympanic with high-pitched bowel sounds present. Rectal examination was normal. Laboratory work was remarkable only for clinically significant leukocytosis (white blood cell count, 16.5 × 10⁹/L). She was admitted for nasogastric tube decompression along with rehydration and replacement of electrolytes. A computerized tomography (CT) scan of the abdomen with intravenous contrast revealed inflammatory changes in the right upper quadrant as well as a high-grade mechanical small bowel obstruction along with intussusception of the small intestine (Figure 1).

Due to the high-grade obstruction, the decision was made to proceed to the operating room. The patient underwent an exploratory laparotomy. There were severe adhesions found in the vicinity of the gallbladder and adjacent duodenum. Exploration revealed massively dilated loops of small bowel proximal to a round, hardened structure within the terminal ileum. A longitudinal enterotomy was performed where a large 5 cm × 3 cm × 4 cm gallstone (Figure 1) was removed. Care was taken to make sure no other stones were present in the small intestine. No segmental small bowel resection was required. The enterotomy was repaired in two layers in a transverse fashion taking care not to create a stricture. The patient tolerated the surgery well and recovered uneventfully. She went home on postoperative day two.

## Discussion

Gallstone ileus is a moderately rare complication of cholelithiasis and a type of mechanical ileus involving obstruction of the intestinal tract due to impaction of a gallstone that has fistulized via a biliary-enteric fistula, most commonly in the duodenum via inflammation of the gallbladder due to acute cholecystitis [1]. Gallstone ileus occurs in 0.3% to 0.5% of all patients with gallstones [2]. Gallstone ileus is the cause of less than 0.1% of all mechanical obstruction cases and occurs in 1% to 4% of non-strangulating mechanical small bowel obstruction [2]. Mortality of gallstone ileus remains high, ranging from 12% to 27%, partially because of non-specific symptoms, unremarkable biochemical investigations, high misdiagnosis rate, older age of patients, late hospital admission, and delayed discovery [2,3]. The diagnosis of gallstone ileus usually requires radiographic studies. An abdominal x-ray can show signs of gallstone ileus and these signs are known as Rigler’s triad, which consists of pneumobilia, small bowel obstruction, and radiopaque gallstone [4]. Because of this, CT is the more commonly used which has been shown to have a sensitivity of 93% [2]. Gallstone ileus can also be diagnosed intraoperatively when a patient is undergoing laparotomy for the unknown origin of small bowel obstruction [2].

Currently, the surgical decisions consist of a simple entero-lithotomy: a one-stage procedure consisting of entero-lithotomy, cholecystectomy, and fistula closure. Another option is a two-stage procedure consisting of an entero-lithotomy and cholecystectomy with fistula closure performed at different times [2]. Location of the gallstone impaction can influence the decision for one or two-stage surgery. For stones impacted at the level of the small intestine, a two-stage surgery is recommended. A one-stage method is recommended for other non-small intestine areas of bowel impaction [1]. However, other factors such as the overall health of the patient and pre-existing conditions can affect the outcome of patients and should be considered by the surgeon, especially when considering a one-stage procedure [1,5]. There is a small risk of recurrence of gallstone ileus managed with a two-stage approach, roughly 5%-9% and of these, only 10% require another operation [6].

## Conclusions

Non-operative methods should be considered in patients that are at higher risk of complications from surgery. As for when conditions do not support the decision of a one-stage procedure, a two-stage surgery should be considered.
